# First-year medical students’ expectations and early experiences of learning critical thinking: a qualitative study

**DOI:** 10.3389/fpsyg.2026.1853293

**Published:** 2026-07-17

**Authors:** Moteeb Alhamidani

**Affiliations:** 1 King Abdullah International Medical Research Center, Riyadh, Saudi Arabia; 2 King Saud bin Abdulaziz University for Health Sciences, Riyadh, Saudi Arabia; 3 Ministry of National Guard—Health Affairs, Riyadh, Saudi Arabia

**Keywords:** critical thinking, learning experience, medical education, qualitative research, student expectations, undergraduate

## Abstract

**Introduction:**

Developing critical thinking (CT) is important in medical education because it supports clinical reasoning and decision-making. However, students may struggle to engage with these skills when students’ learning expectations are unclear or inconsistent with their educational experiences. Understanding how students’ expectations shape their early learning encounters is important for supporting successful transition into medical education. This study explored first-year medical students’ expectations of learning critical thinking and how those expectations were reflected in their subsequent experiences in a science course.

**Methods:**

A qualitative descriptive design, situated within an interpretivist epistemology, was employed. Seven first-year undergraduate medical students at a medical school were recruited through purposive sampling and interviewed individually. Data were analysed using reflexive thematic analysis.

**Results:**

Four themes were identified: (1) expectations before entering the course, (2) early encounters with course reality, (3) alignment and misalignment between expectations and experience, and (4) shifting interpretation of critical thinking over time. Students expected critical thinking to involve analytical reasoning and problem-solving. Early course experiences either confirmed or challenged these expectations. When expectations and experiences diverged, students reported uncertainty about course aims. Over time, students actively reinterpreted what critical thinking meant in their specific course context.

**Discussion:**

The findings suggest that expectations appeared to operate as interpretive filters, shaping how students engaged with early learning activities, and that clarity about learning aims reduces uncertainty during early course engagement.

## Introduction

Critical thinking (CT) is consistently identified as a core competency in medical education, essential for clinical decision-making and professional practice (Zainal et al., 2025; Shakurnia et al., 2021; Mukhopadhyay and Choudhari, 2024). For the purposes of this study, CT is understood as a purposeful, self-regulatory process of interpretation, analysis, evaluation, and inference, used to guide belief and action in clinical and educational contexts, consistent with CT constructs applied across health professions education (Zainal et al., 2025; Shakurnia et al., 2021). Students who develop these skills demonstrate the ability to analyse data, evaluate evidence, make sound decisions, and reflect critically on their own reasoning. Conversely, students with underdeveloped CT skills may struggle with clinical reasoning and patient problem-solving (Zainal et al., 2025; Mukhopadhyay and Choudhari, 2024).

In this study, “expectations” refers to students’ anticipations of what CT learning will involve: the types of tasks they will be asked to perform, the degree of structure or open-endedness they expect, and the role they expect the teacher to play. These expectations are shaped by prior educational experiences and by how CT is framed in institutional messaging (Tan et al., 2025). Schema theory provides the interpretive lens for this study: prior knowledge structures guide how new information is processed, and students therefore evaluate new learning activities through the filter of what they have previously encountered (Rumelhart, 1980; McVee et al., 2005). Accordingly, two students in the same course may assign fundamentally different meanings to the same activity depending on the expectations they bring to it.

CT has been measured in health professions education primarily through two approaches: skill-based instruments and disposition inventories. Skill-based tools, such as the Watson-Glaser Critical Thinking Appraisal and the California Critical Thinking Skills Test (CCTST), assess the ability to analyse arguments, evaluate inferences, and draw conclusions (Azizi-Fini et al., 2015; Shakurnia et al., 2021; Watson and Glaser, 1980; Ross et al., 2013). Disposition inventories, such as the California Critical Thinking Disposition Inventory (CCTDI), measure students’ motivation and tendency to engage in CT rather than their demonstrated ability (Shakurnia et al., 2021; Köksal, 2020). Both approaches treat CT as an outcome variable, measuring levels at defined points in training or comparing groups across instructional conditions (Azizi-Fini et al., 2015; Dehghanzadeh and Jafaraghaee, 2018; Bilik et al., 2020). What these approaches do not capture is how students interpret CT as a learning aim in the first place; the interpretive process that precedes any measurable outcome.

While this study adopts a working definition, students entering medical education do not arrive with a shared understanding of CT; the term is used in diverse and sometimes inconsistent ways across educational contexts (Wechsler et al., 2018; Châlon and Lutaud, 2024). Although CT is frequently highlighted in course descriptions and curricular frameworks in health professions education (Shakurnia et al., 2021; Kahlke and Eva, 2018), different instructional traditions emphasise different facets of the construct. Some educators associate CT with problem-solving, others with questioning assumptions, and others still with applying knowledge in unfamiliar situations (Shakurnia et al., 2021). Students therefore enter courses with differing interpretations of what learning CT will involve, a direct consequence of the definitional inconsistency documented in the literature (Wechsler et al., 2018; Châlon and Lutaud, 2024).

Students’ expectations of learning are shaped by prior educational experiences and institutional messaging (Tan et al., 2025). Because students arrive from varied instructional backgrounds, they enter training with different CT skill levels (Hunter et al., 2014) and respond differently to CT-focused activities (Dehghanzadeh and Jafaraghaee, 2018). When their expectations diverge from course activities, students may experience uncertainty and reduced confidence, responses linked to stress in health professions students (Jang and Park, 2020; Akkaya et al., 2018).

Despite the recognised importance of CT in early medical training, few studies have directly examined how first-year medical students’ expectations of learning CT compare with their subsequent experiences within a specific course context, or how students interpret CT as a learning aim during early course exposure. This study aimed to explore first-year medical students’ expectations of learning critical thinking and to describe how those expectations compared with their early learning experiences in a science course.

## Theoretical framework

This study was guided by schema theory, which provides both the conceptual lens for understanding student expectations and the analytic orientation for the study design. Schema theory holds that knowledge is organised into mental structures, schemas, built from prior experience, and that these structures guide how individuals perceive, interpret, and assimilate new information (Rumelhart, 1980; McVee et al., 2005). When learners encounter a new situation, they do not process it neutrally; they interpret it through whichever schema their prior experience activates, and this determines which features they attend to, what meaning they assign, and what inferences they draw (Rumelhart, 1980). The same activity can therefore be understood in fundamentally different ways by different learners, depending on the schema each brings to it.

Within this framework, students’ expectations of learning CT are understood as schema-derived anticipations: the educational schemas students have built from prior experience lead them to expect CT learning to take a particular form, particular kinds of tasks, a particular degree of structure, and a particular role for the teacher. These expectation-based schemas operate as interpretive filters during early course engagement. When a course activity fits the activated schema, it is readily assimilated and recognised as CT, producing a sense of alignment; when an activity does not fit, students must either restructure their schema to accommodate the new experience or remain uncertain about whether the activity constitutes CT as intended (Rumelhart, 1980; McVee et al., 2005). Schema theory thus accounts for both the variation in how students interpret identical activities and the ongoing interpretive adjustment that occurs as they accumulate course experience.

This framework guided the study at three levels. It informed the design: because schema theory locates meaning in the learner’s interpretive process rather than in measurable outcomes, an interpretivist qualitative approach centred on students’ own accounts was required. It shaped data collection, as the interview guide was structured to surface students’ prior schemas (their expectations before the course), the testing of those schemas against early activities, and any subsequent restructuring of understanding. And it oriented the analysis, sensitising the reflexive thematic analysis to the alignment and misalignment between expectations and experience, and to the interpretive shifts over time that became central to the findings. Conceptualising expectations as schema-based interpretive filters in this way distinguishes the present study from outcome-focused CT research and foregrounds the meaning-making that precedes any measurable change in CT skill.

## Methods

### Study design

This study employed a qualitative descriptive design, situated within an interpretivist epistemology. Interpretivist research holds that meaning is constructed through individual experience and that the researcher’s role is to understand and represent those constructions faithfully (Lincoln and Guba, 1985). A qualitative descriptive design was chosen because it aims to describe phenomena from the perspectives of those who experience them, without imposing theoretical frameworks or seeking causal explanations, an approach well suited to exploring student perceptions and interpretations in educational settings (Sandelowski, 2000; Neergaard et al., 2009). Data were analysed using reflexive thematic analysis following the phases outlined by Braun and Clarke (2006, 2019, 2022, 2023), which allows for exploration of how students interpret and make sense of their learning experiences.

### Setting and participants

The study was conducted at a medical school. The science course in question was a first-year compulsory course that explicitly stated CT as a central learning aim. The course involved laboratory work, scientific literature review, and case-based activities. First-year undergraduate medical students were selected because they were at the beginning of their training, had limited prior exposure to medical education conventions, and were therefore well placed to reflect on early expectations and experiences.

A purposive sampling strategy was used to recruit students who could provide information-rich accounts of their expectations and early course experiences (Malterud et al., 2016). Students were contacted via a course administrator who distributed study information sheets by email; the researcher had no teaching role in the course at the time of recruitment. According to Malterud et al. (2016) information power framework, a smaller sample may be sufficient when the study aim is narrow and specific, participants are well positioned to address that aim, and the analysis is theory-driven and dialogic, all of which applied here. Specifically, the aim was narrow (students’ expectations and early experiences of CT in one course), participants were uniquely positioned to address that aim (enrolled first-year students, interviewed mid-semester), and the analysis was theory-driven and dialogic in line with Braun and Clarke’s reflexive approach. Seven first-year undergraduate medical students were recruited midway through the academic year, allowing time for initial impressions to form while initial expectations remained accessible to recall. Students were included if they were enrolled in the target course and agreed to participate voluntarily. Students who chose not to participate after receiving the study information, or who withdrew before completing their interview, were excluded; no students withdrew in practice. Demographic detail was minimised to preserve confidentiality given the small sample. Participants ranged in age from 18 to 20 years. All had completed secondary education in Saudi Arabia prior to enrolment.

### Data collection

Semi-structured interviews were conducted with each participant individually. All interviews were conducted by the researcher, a medical education researcher with prior training in qualitative interviewing and reflexive thematic analysis. The researcher had no prior teaching, supervisory, or assessment relationship with any participant. Participants were informed that participation was voluntary, independent of course assessment, and would have no effect on their academic standing. Only a single interview was conducted with each participant. Interview topics included expectations before the course, early impressions of learning activities, experienced teaching and learning activities, and perceived alignment or mismatch between expectations and experiences. The interview guide was designed to elicit open-ended accounts and covered five topic areas: (1) prior understanding of CT before the course; (2) initial reactions to course activities; (3) specific activities experienced as CT or not CT; (4) moments of clarity or confusion about course aims; and (5) how understanding of CT shifted over time. Interviews were audio-recorded with participant consent and transcribed verbatim. Each interview lasted between 30 and 45 min. Data collection was completed within a single semester.

### Data analysis

Reflexive thematic analysis was conducted following Braun and Clarke’s six phases: familiarisation with the data, generating initial codes, constructing themes, reviewing themes, defining and naming themes, and producing the written analysis (Braun and Clarke, 2006, 2019). The first author led the coding process, generating initial codes inductively from all seven transcripts. Initial codes were grounded in participants’ own language and captured both manifest and latent content. The analytical team, comprising the author and one independent researcher with backgrounds in medical education and qualitative inquiry, reviewed and discussed codes iteratively until consensus was reached on theme construction. Where initial interpretations diverged, the team returned to the raw transcript data and discussed the coding decision until agreement was reached through collaborative reasoning. Themes were understood as patterns of shared meaning organised around a central concept, consistent with Braun and Clarke’s reflexive approach. No claim of data saturation was made; instead, analytic sufficiency was evaluated in relation to the study’s focused aim and the information-richness of the sample (Malterud et al., 2016).

### Ethical considerations

Ethics approval was obtained from the institutional review board of King Abdullah International Medical Research Center (KAIMRC). All participants provided written informed consent prior to the interview. Confidentiality was maintained through anonymisation of all identifying information in transcripts and reporting. Participants are identified in the findings using anonymised codes (P1–P7).

## Results

Four themes were identified from the interview data. These themes describe the students’ expectations before entering the course, their early encounters with course reality, the alignment and misalignment between expectations and experience, and their shifting interpretation of critical thinking over time. Each theme is presented below with supporting participant accounts.

### Theme 1: Expectations before entering the course

Students did not enter the course as blank slates; they carried specific and varied prior frameworks that actively shaped how they approached early learning activities. Most (five of seven) expected CT to centre on analytical reasoning and problem-solving: evaluating evidence, applying knowledge to unfamiliar situations, or identifying weaknesses in arguments. Three participants described anticipating activities resembling those they had encountered in secondary education.

*“I thought critical thinking would be about picking apart arguments and finding flaws, like we did in essay writing at school, so I expected a lot of discussion and debate.” (P3)*

*“I assumed we would be doing things like analysing patient cases, working through what the evidence meant for a diagnosis. I thought that was what critical thinking in medicine would be.” (P7)*

Two participants connected CT more closely to scientific reasoning or clinical problem-solving, associating it with the diagnostic and evaluative demands they expected medical education would eventually place on them.

Across all seven participants, there was no shared prior understanding of what CT activities in this course would specifically look like. These differences in prior understanding shaped how students initially approached course activities.

### Theme 2: Early encounters with course reality

When students engaged with the course for the first time, their reactions reflected how closely early activities matched what they had anticipated. Three participants found that early tasks confirmed their expectations.

*“The tasks in the first few weeks were exactly what I thought critical thinking would be, reading articles, finding the gaps, questioning the conclusions. It matched my expectations very well.” (P6)*

Students who had expected CT to involve evaluating scientific data reported that activities requiring interpretation of research findings were consistent with what they had imagined.

*“When we had to read a journal article and identify what the study was actually claiming versus what the evidence showed, that felt like exactly what I thought critical thinking was.” (P1)*

For four participants, early encounters challenged their expectations. These students expressed surprise at activities presented as CT that they did not immediately recognise as such. One described uncertainty about whether group discussions constituted CT or simply collaborative work. Another noted that activities felt more structured than anticipated, with less room for open-ended questioning.

*“I expected more freedom to just ask questions and explore, but a lot of it was following a specific framework or answering set questions. I wasn’t sure at first if that was what they meant by critical thinking.” (P5)*

In these early encounters, students compared what they observed in the course against what they had anticipated CT to look like. The variation in first impressions reflected how closely each student’s prior understanding matched the course’s actual approach.

### Theme 3: Alignment and misalignment between expectations and experience

As students progressed through early weeks of the course, four of seven encountered activities that did not match what they had anticipated CT to involve.

Misalignment occurred when course activities differed from what students had imagined. Four of seven participants reported some degree of mismatch. Those who had expected debate and discussion found activities were more framework-guided than anticipated. Two who had expected independent inquiry found tasks to be more directed.

*“I kept waiting for the moment where we’d just be let loose to think for ourselves, but the tasks always had a clear structure. After a while I started wondering whether I was misunderstanding what critical thinking was supposed to mean in this course.” (P6)*

Misalignment was not uniformly described as negative. Two students reflected that encountering an unexpected approach prompted them to reconsider their own assumptions about what CT involves. However, for most, mismatches created a period of uncertainty about whether they were engaging with CT as intended. One participant (P7) reported no strong sense of misalignment, describing the course as broadly consistent with what they had expected, a reminder that the pattern of mismatch was not universal.

### Theme 4: Shifting interpretation of critical thinking over time

A striking feature of the interview accounts was the ongoing, incomplete nature of students’ interpretive work. Across the semester, students did not settle on a fixed understanding of CT; instead, they continually revised their interpretation as they accumulated course experience. This was not a linear process of progressive development but an ongoing adjustment driven by repeated engagement with specific activities.

Most participants (five of seven) described a broadening of their understanding, coming to recognise that CT could include reflection, self-questioning, and attention to process, as well as analytical reasoning.

*“By the middle of the semester I started to realise that questioning my own reasoning, not just the material, was part of it too. That wasn’t what I expected at the start.” (P2)*

Two participants maintained a narrower, more course-specific interpretation, focusing on the techniques emphasised in class. Across both groups, five of seven reported that uncertainty about CT did not disappear entirely, particularly regarding how CT in this science course connected to clinical practice or other areas of their medical education.

*“I think I understand what they want in this course now. But I’m still not sure how it connects to what we’ll need to do as doctors. That part is still a bit vague to me.” (P4)*

Uncertainty about CT’s relevance to clinical practice persisted across most participants by the end of the semester, even where course-level understanding had improved.

## Discussion

This study explored how first-year medical students’ expectations of learning CT were reflected in their early course experiences. The findings contribute a student-centred, interpretive account of CT pedagogy in early medical education, a perspective that is distinct from the predominantly outcome-focused literature (Araújo et al., 2024; Ho et al., 2023; Manuaba et al., 2022) and from studies that measure CT skill levels without attending to how students understand the construct in the first place.

The central finding of this study is that expectations functioned as interpretive filters, determining which features of a learning activity students attended to, how they assigned meaning to those features, and whether they judged themselves to be engaging with CT as intended. This finding builds on schema theory, which proposes that prior knowledge structures guide how new information is processed and interpreted (Rumelhart, 1980). Recent research has similarly shown that medical students’ expectations function as implicit contracts that shape their engagement with institutional programmes (Tan et al., 2025). Students did not encounter course activities as neutral events; they evaluated them through the lens of what they had anticipated CT to be. This is consistent with observations in health professions education that CT frameworks developed through training shape professional practice (Zainal et al., 2025; Shakurnia et al., 2021), and with findings in nursing education showing that different instructional approaches produce different patterns of CT engagement (Dehghanzadeh and Jafaraghaee, 2018). Research in nursing education has found a positive relationship between CT skill level and self-efficacy (Orujlu and Hemmati Maslakpak, 2017), suggesting that how students engage with CT has consequences for confidence in practice.

When expectations and experiences aligned, students reported clearer understanding of learning aims and more purposeful participation. When they diverged, students experienced uncertainty about whether they were meeting course intentions. This finding is consistent with evidence from health professions education that misalignment between student expectations and course realities can affect students’ sense of direction in their learning (Jang and Park, 2020; Tan et al., 2025). Importantly, misalignment was not uniformly negative: for some students, encountering unexpected approaches led them to reconsider their prior assumptions about CT. This nuance suggests that the pedagogical aim should not be to eliminate misalignment entirely, but to ensure that students have the contextual clarity needed to make sense of it when it occurs.

The fourth theme, shifting interpretation of CT over time, highlights the interpretive work students perform throughout a course. This work is rarely acknowledged in CT curricula, which tend to focus on skill acquisition rather than on how students make sense of the construct itself (Batarfi and Agha, 2025). The finding that students’ understanding remained partially uncertain, particularly regarding the connection between CT in this course and CT in clinical practice, points to a gap in how early CT education is framed. Students in this study were engaged in a learning process whose broader purpose remained opaque to them. In this study, students who gained clearer understanding of CT aims reported more purposeful engagement with course activities, consistent with the broader principle that clarity about learning expectations reduces uncertainty and stress in health professions training (Tan et al., 2025; Anderson and Fernandez-Branson, 2024).

This study’s contribution to the medical education literature is threefold. First, it foregrounds the interpretive work first-year students perform before any change in CT understanding or skill can be observed. Second, it demonstrates that this interpretive work is both active and incomplete, students continuously revised their interpretation of CT across the semester rather than arriving at a settled definition of CT, pointing to the ongoing nature of CT meaning-making in early training. Third, it identifies clarity about learning aims, not uniformity of interpretation, as the educational condition most likely to support productive engagement with CT in early medical training. These contributions distinguish this study from prior work that treats CT as an outcome to be measured rather than a construct to be understood from the learner’s perspective.

## Implications for practice

First, the diversity of expectations students reported on entry (Theme 1) suggests that CT should not be assumed to be a shared concept. Course inductions and orientation sessions should include an explicit discussion of what CT means in the specific course context, ideally with concrete examples of activities that do and do not constitute CT as defined in that course. This does not require definitional uniformity; rather, it provides students with an interpretive anchor from which to work.

Second, the uncertainty students experienced during periods of misalignment (Theme 3) suggests that making the rationale for learning activities visible could reduce interpretive confusion. When introducing activities that students might not recognise as CT, educators can briefly explain how the task relates to CT aims, for example, by naming the specific CT skill the activity is designed to develop (Anderson and Fernandez-Branson, 2024; Manuaba et al., 2022). This small intervention may significantly reduce the period of unproductive uncertainty described by participants.

Third, the ongoing, incomplete nature of students’ interpretive reframing (Theme 4) suggests that CT learning aims should be revisited throughout the course, not only stated at the outset. Structured opportunities for students to reflect on how their understanding of CT has shifted, for example, through brief written reflections at course midpoint, may help students recognise and articulate their own developing understanding.

Finally, the persistent uncertainty about how CT in this course connected to clinical practice points to a structural gap in how early CT education is framed. CT has been shown to directly influence clinical decision-making quality (Dewi et al., 2021; Mukhopadhyay and Choudhari, 2024); making this connection explicit in early training may help students understand why CT development matters and engage with it more purposefully.

## Limitations

Several limitations should be considered. The sample was small (*n* = 7) and drawn from a single institutional context, which limits transferability (Malterud et al., 2016; Lincoln and Guba, 1985). All interviews were conducted by the primary researcher, which may have introduced an interviewer effect despite the reflexive strategies described above.

Data were collected at a single point in the semester through retrospective interview, meaning that participants’ accounts of their initial expectations may have been shaped by subsequent course experiences and memory reconstruction. The study relied on self-reported perceptions rather than observed behaviours or measured outcomes. Additionally, factors outside the course, including other concurrent courses, personal circumstances, or prior educational systems, may have influenced how students experienced and interpreted CT. Future research would be strengthened by longitudinal designs that collect data at several points across the semester rather than at a single time point, which would give a deeper, less retrospective account of how students’ interpretations of CT evolve and would strengthen the reliability of the patterns identified here. Replicating the study in different institutional and disciplinary settings, with larger and multi-site samples, would further test these findings and enhance their transferability and generalisability.

## Conclusion

This study examined how first-year medical students’ expectations of learning CT shaped their early encounters with a science course that explicitly emphasised CT as a learning aim. Four themes traced a dynamic process from entry expectations, through early encounters and interpretive mismatches, to the active reinterpretation of what CT meant in practice.

The study’s central contribution is an account of CT learning as an interpretive process, not only a skill acquisition process. Students did not passively receive a definition of CT and then apply it; they actively worked to make sense of an abstract aim through the filter of their prior expectations. This interpretive work, which is rarely acknowledged in CT curricula, has real consequences for student engagement, particularly during the critical early weeks of a course.

For educators, the practical implication is clear: making CT aims explicit, contextually grounded, and connected to their clinical purpose is not a peripheral concern; it is foundational to the quality of early CT education. Students who understand what they are being asked to develop, and why it matters, are better positioned to engage productively with learning activities, even when those activities challenge their initial expectations.

More broadly, this study contributes a student-centred lens to the discourse on CT pedagogy in early medical education (Kahlke and Eva, 2018; Châlon and Lutaud, 2024; Ho et al., 2023), foregrounding the interpretive work students perform before any skill development can be measured. Future research should examine how explicit curriculum framing of CT aims affects not only student reported understanding but also subsequent CT engagement and learning outcomes over time.

## Data Availability

The raw data supporting the conclusions of this article will be made available by the authors, without undue reservation.
